# The gut-microbiota-brain axis in autism: what *Drosophila* models can offer?

**DOI:** 10.1186/s11689-021-09378-x

**Published:** 2021-09-15

**Authors:** Safa Salim, Ayesha Banu, Amira Alwa, Swetha B. M. Gowda, Farhan Mohammad

**Affiliations:** Division of Biological and Biomedical Sciences (BBS), College of Health & Life Sciences (CHLS), Hamad Bin Khalifa University (HBKU), Doha, 34110 Qatar

**Keywords:** Gut-microbiome-brain axis, Autism spectrum disorders, Gastrointestinal issues, *Drosophila*

## Abstract

The idea that alterations in gut-microbiome-brain axis (GUMBA)-mediated communication play a crucial role in human brain disorders like autism remains a topic of intensive research in various labs. Gastrointestinal issues are a common comorbidity in patients with autism spectrum disorder (ASD). Although gut microbiome and microbial metabolites have been implicated in the etiology of ASD, the underlying molecular mechanism remains largely unknown. In this review, we have summarized recent findings in human and animal models highlighting the role of the gut-brain axis in ASD. We have discussed genetic and neurobehavioral characteristics of *Drosophila* as an animal model to study the role of GUMBA in ASD. The utility of *Drosophila* fruit flies as an amenable genetic tool, combined with axenic and gnotobiotic approaches, and availability of transgenic flies may reveal mechanistic insight into gut-microbiota-brain interactions and the impact of its alteration on behaviors relevant to neurological disorders like ASD.

**Supplementary Information:**

The online version contains supplementary material available at 10.1186/s11689-021-09378-x.

## Introduction

The human body hosts ~100 trillion microbial species on the skin, respiratory tract, genitals, etc. However, the majority of microbial species reside in the gut [1]. The gut microbiome (GM) comprising commensal, symbiotic, and pathogenic microbial communities influences host physiology and pathophysiology such as immunity and metabolism [2]. The GM can respond to stress and brain injuries like traumatic brain injury and ischemia by modulating its composition [3]. The GM interacts with the host system and may affect host homeostasis by affecting nutrient processing, availability, and absorption [1].

**Fig. 1:**
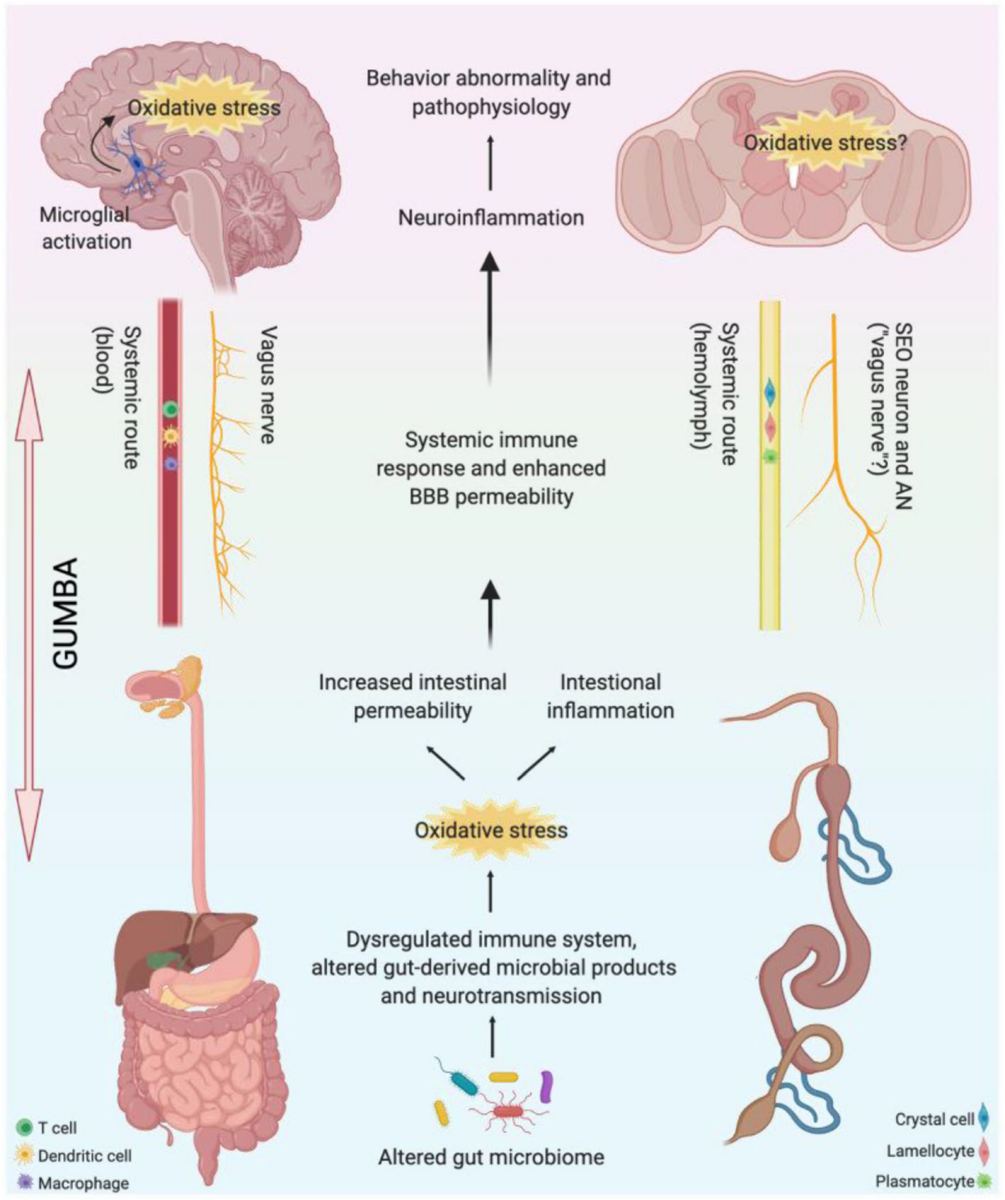
Illustration of GUMBA in development of neuropathology in humans and fruit flies. Changes in the GM composition and resultant altered gut-derived microbial products and neurotransmission can over-activate the immune system producing increased oxidative stress. Sustained oxidative stress can increase intestinal permeability and inflammation, thus triggering systemic inflammation, which can enhance the blood-brain barrier permeability and cause microglial activation in the brain through the transport of immune cells and metabolites via the systemic route. This would, in turn, enhance oxidative stress and exacerbate neuroinflammation in the brain, resulting in disease pathophysiology and abnormal behavior. Changes in GM composition and its metabolites can also alter vagus nerve signaling and exert effects on the brain and behavior. Many organ systems are homologous between humans and flies; however, the mechanistic basis of GUMBA involvement in neuropathology development remains to be uncovered in flies. SEO, serotonergic output; AN, antennal nerve (Created with BioRender.com)

The gut-microbiota-brain axis (GUMBA) is a bidirectional communication between the GM and the brain with multiple routes and mechanisms, including neural, endocrine, and immune pathways [4, 5]. In humans, the neural pathway consists of the vagus nerve that plays both afferent and efferent roles in communication between the central nervous system (CNS) and enteric nervous system (ENS) [6, 7]. The GM stimulates the afferent neurons of the ENS, which sends signals to the brain via the vagus nerve that innervates the proximal colon and intestine [8]. The GM can also release neurotransmitters like acetylcholine, gamma-aminobutyric acid, adrenaline, serotonin, dopamine, or neuroactive molecules like short-chain fatty acids (SCFAs) that can induce changes in CNS [8] via the endocrine pathway. The neurotransmitters and neuroactive molecules released by the GM can be grouped under the neuronal pathway considering their pronounced effect on the CNS (Fig. 1). Changes in the gut microbial composition can lead to alteration in levels of metabolites such as SCFAs and lipopolysaccharides. This can compromise the gut metabolism and trigger enhanced immune response and mitochondrial dysfunction, resulting in increased oxidative stress. Sustained oxidative stress can in turn increase intestinal permeability and inflammation, which can exert pro-inflammatory activity and enhance blood-brain barrier permeability. Microglial activation in the brain can further exacerbate inflammation resulting in malfunction of synapses and manifesting as behavioral abnormalities and neuropathologies [9–14]. Similar to the mammalian system, in *Drosophila*, altered GM may also result in epithelial oxidative burst causing changes in gut permeability and affecting longevity and behaviors [15–18] (Fig. 1).

Autism spectrum disorder (ASD) is a group of complex developmental disorders clinically characterized by behavioral symptoms, such as impaired communication skills, defective social interaction, and the presence of repetitive behaviors [19]. ASD significantly limits the individual’s capacity to live independently, posing a substantial burden on their families. These disorders represent a serious public health concern as their global prevalence has increased with improved detection methods [20]. Although ASD is primarily considered to be a neurodevelopmental disorder, it does not involve only the brain: the gut as well as an interplay between genetic and environmental factors like the microbiome has a crucial role in autism etiology [20–22]. A broad range of gastrointestinal (GI) symptoms, such as diarrhea, constipation, abdominal pain, bloating, food allergies, vomiting, gaseousness, and foul-smelling stools, are four times more prevalent in children with ASD in comparison to healthy children [11, 23–25]. However, it remains unclear if defective GUMBA plays a causative role or leads to only comorbidities [26].

The primary support to the hypothesis of GUMBA involvement in ASD comes from various evidence, including gut microbiology, microbial endocrinology, and behavioral studies. A recent study on a rodent autism model constituting maternal obesity and high-fat diet (MHFD) offspring showed that GM dysbiosis mediated the social behavior deficits [27]. Social behavior defects in MHFD mice were restored upon recolonization of microbiota from regular mice in germ-free mice. Moreover, selective recolonization of the mice gut with *Lactobacillus reuteri* reversed the defects in social behavior [27]. *L. reuteri* microbial intervention was also recently shown to reverse the social deficits in Cntnap2−/− mouse model of ASD by improving the social reward, perhaps through upregulation of metabolites in the tetrahydrobiopterin pathway [28]. These studies provide strong support for the bidirectional communication hypothesis between the gut and the brain in ASD.

While it is known that the composition and diversity of the GM may depend on the host genetic background [29–31], it is unknown whether there is a signature microbiome that is maximally influenced in an ASD background. Furthermore, the molecular mechanism that regulates host-microbiota homeostasis in normal and ASD states is not known. The analyses needed to address this knowledge gap are challenging to perform in humans and rodents due to the high costs and tedious process of comparing the microbiomes of multiple genetic models of ASD.

*Drosophila melanogaster* can aid in GUMBA research by providing advanced genetic and genomic toolkits to manipulate all cell types in the gut and the brain, along with tractable microbiome systems. At the genetic level, around 65% of *Drosophila* genes are similar to human genes, and 75% of human disease genes have orthologs in fruit flies [32, 33]. Most of these genetic homologs are expressed in *Drosophila* tissues and perform functions equivalent to those in human tissues [34]. A number of ASD genes have been functionally characterized using *Drosophila* for their impact on ASD-related behaviors like intellectual disability and social interactions [34–39], thus further establishing *Drosophila* as a valuable model to study mechanisms underlying human diseases [39]. In a recent study, Chen et al. [36] showed that genetic variation in the protein encoded by one of the autism gene orthologs in *Drosophila*, lysine demethylase 5 (KDM5), leads to changes in gut physiology, microbiota composition, activation of immune deficiency (IMD) pathway, and social interaction behavior. However, apart from social interaction, whether KDM5 effects on GUMBA also affect other ASD core features remains unclear. Similar to KDM5, many of the evolutionarily conserved ASD genes (Supplementary Table 1) can further be studied in much more detail to describe their effects on the microbiota and ASD-related behaviors. Genetic mutant or cell-specific knockdown of the candidate ASD genes in *Drosophila* intestine could be used to study whether the *Drosophila* GM changes in response to genetic backgrounds [36]. The *Drosophila* system can further be studied to answer if changes in expression of ASD genes in the *Drosophila* gut leads to microbiome changes causing induction in oxidative stress, and alteration in ASD-related behaviors (Fig. 1).

## Increasing prevalence of GI issues in ASD patients: evidence for altered gut-brain axis

Among several comorbidities, GI distress in patients with ASD has recently gained attention mainly because of its prevalence and severity [40]. Although there is no direct evidence that GI problems are causative of ASD, multiple studies have suggested that the altered interactions of the GM and the brain might play an important role in ASD physiology. As mentioned earlier, ASD is associated with a range of chronic GI symptoms, including altered bowel habits, abdominal pain, and food intolerance [37]. Gut distress or abdominal discomfort can give rise to behavioral difficulties and potentiate ASD’s core stereotypical behaviors, like mouthing gestures, screaming, and self-injury to the abdomen to relieve pain [35]. However, because of communicative defects in ASD patients, GI issues often remain undiagnosed and hence untreated. The prevalence of GI issues in children with ASD is reported to be 23–90% [38, 41–44]. The risk of malignancy, specifically for colon cancer, also seems to be elevated in affected patients [45]. However, GI issues are more prevalent in those patients that show all the key features of autism rather than just a subset of core features [46], suggesting that GI issues are core features of ASD.

## GM is a critical component of the gut-brain axis, but its association with ASD is unclear

Although the role of GM in relation to human health and disease has been extensively explored over the past decade, its relationship with ASD has only recently attracted attention. Several studies have reported that individuals with ASD and GI issues exhibit an altered gut microbial composition [47–50]. Furthermore, a recent meta-analysis of nine studies involving patients with ASD identified specific microbial species altered in children with ASD, specifically *Bifidobacterium*, *Bacteroides*, and *Lactobacillus* species [51, 52]. However, the correlation between patient genetic information and the GM profile has not been thoroughly explored. Interestingly, antibiotic treatment can improve ASD symptoms in some children [53–55]. Although these findings support the idea of the role of the gut and its microbiome in mediating ASD, could it also suggest that ASD individuals are prone to bacterial infections? More studies are required to answer this question. Moreover, several animal model studies have implicated microbiota involvement in models of stress, anxiety, depression, and autism [36, 45, 56–58]. Interestingly, around 60% of patients with significant mood or affective or psychiatric disorders are thought to have a functional GI disorder [41].

The genetic factors that lead to ASD are not fully understood. ASD is highly heritable [59], but the genetic basis is exceedingly complex, and any single gene that has been associated with ASD accounts for less than 1% of cases [60]. Multiple studies have identified several hundreds of human genes associated with ASD [61] in scientific efforts to understand the molecular mechanisms underlying ASD worldwide. However, because of high costs, ethical issues, and long animal life cycles, conventional approaches have provided limited insight into the potential functional relevance of these gene candidates or the molecular mechanisms underlying ASD. What is needed is a rapid and effective method to screen conserved gene candidates for functional outcomes and their validation in physiologically relevant mammalian systems.

## *Drosophila* serves as a robust model to study the gut-brain axis and its role in ASD

Over the last two decades, genome-wide association studies (GWAS) or whole genome or exome sequencing (WGS/WES) have revealed thousands of genetic risk factors associated with various human disorders [62]. However, despite the impressive success rate of finding disease susceptibility loci, the clinical insights derived from these results have been limited, and very few potential loci have successfully been functionally validated [62, 63]. Hence, the biological context of a large number of identified risk loci remains unclear.

Owing to the vast array of tools for gene manipulation, availability of a large number of insertion or deletion alleles for the majority of fruit fly genes, including homologs of candidate susceptibility genes from GWAS or WES/WGS, and ease of doing experiments with large sample size, *Drosophila*-based studies have robustly touched almost every human disease biology [63, 64]. Although there are many differences between fruit flies and humans, significant similarities between vertebrates and flies have been demonstrated at the physiological, molecular, and genetic levels [32, 65, 66]. Many vertebrate genes have orthologs expressed in corresponding *Drosophila* tissues, which perform functions equivalent to those in human tissues [34, 67]. A recent study identified 91 novel ASD-associated genes by analyzing de novo mutation burden in coding regions of candidate genes. Using light-off jump habituation assays in fruit flies, the study functionally characterized the novel genes and found that ~40% of genes showed a defect in suppressing startle response [39]. This indicates that a large proportion of ASD-associated genes have a measurable ASD-related phenotype in *Drosophila*. Also, apart from learning phenotypes, a few ASD genes have also been shown to regulate *Drosophila* social behavior by impacting the GM [36].

### *Drosophila* vagus nerve

In vertebrates, the vagus nerve is a major cranial nerve, which travels from the brain and connects to the intestine. In *Drosophila* larva, serotonergic output (SEO) neuron leaves CNS via the antennal nerve (AN), analogous to the vertebrate vagus nerve [68, 69]. The SEO neuron in *Drosophila* larva innervates central endocrine system median neurosecretory cells (mNSCs) and the ENS and carries sensory information from various body organs like the olfactory ring gland, pharyngeal, and other internal organs, including the intestine [69, 70]. However, it is unknown whether the serotonergic antennal nerve (AN) sustains the metamorphosis stage and functions in adult flies as the primary “vagus nerve” and innervates the intestine.

## *Drosophila* GM research offers several advantages for analyzing gene-microbiome-brain interactions

The most direct evidence in establishing a causal relationship between gut-microbiome and ASD that distinguishes it from effect mediated by genetic variants has come from studies in rodents. Different approaches for introducing pre-and post-natal alterations in the microbiome and microbial metabolites perturb various social behaviors associated with neurodevelopmental disorders, including ASD, both in humans and mouse models (reviewed in [71]). For instance, a germ-free humanized mouse model colonized with microbiota from ASD patients promoted ASD-like features, such as decreased communication, increased repetitive behavior, social behavior deficit, and decreased locomotion in an open field [28, 72].

Genetically or environmentally manipulated rodent models of autism have furthered our understanding of molecular mechanisms through which microbial dysbiosis in ASD affects animal behaviors [73]. Using known mouse models of ASD (e.g., MHFD), researchers can apply top-down approaches to study if the ASD model possesses microbial dysbiosis and if the altered microbiome contributes to the behavioral deficit. Bottom-up approaches (germ-free, gnotobiotic, antibiotic, and probiotics) allow researchers to study the GM’s immunological, physiological, and behavioral aspects [73].

Compared to the high level of diversity in the mammalian gut (>500 taxa), *Drosophila* has a simple microbiome comprising only 5–30 taxa [52, 74]. The *Drosophila* microbiome is most frequently associated with *Lactobacillus, Acetobacter*, *Enterococcaceae*, and to a lesser extent with *Wolbachia* [51, 75]. *Drosophila* microbiome affects gut physiology and homeostasis, including gene expression, metabolism [76], anxiety-related behavior, sleep [77], and social behavior [36, 78].

**Fig. 2:**
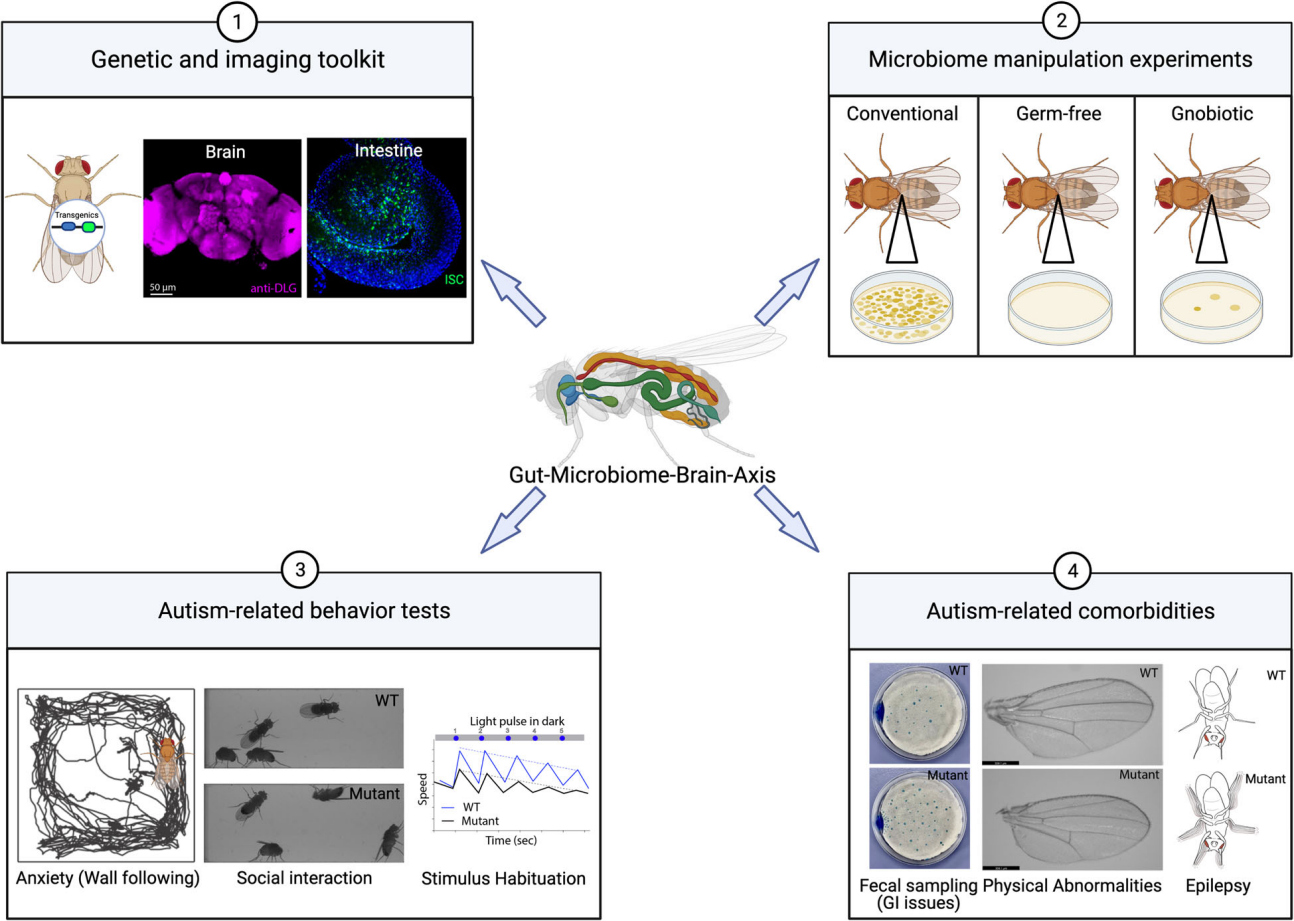
Advantages of using *Drosophila* to study GUMBA in autism. (1) Transgenic flies can be generated to overexpress or knockdown genes of interest in the whole organism or specific cells. The expression of proteins can be easily visualized using immunohistochemistry. The effect of gene knockdown on brain development can also be analyzed by imaging techniques. (2) Flies that are microbiologically sterile (germ-free) or bear microbiota of interest (gnotobiotic) can be readily generated and maintained in large numbers. (3) and (4) In flies obtained from genetic manipulation or microbiome manipulations, autism-related behavioral aspects such as anxiety, social interaction, and stimulus habituation, and comorbidities such as gastrointestinal issues (fecal sampling), physical abnormalities, and epilepsy, can be effortlessly studied using simple, optimized, and robust assays (Created with BioRender.com)

Microbiome research in *Drosophila* is aided by advanced genetic and genomic toolkits and tractable microbiome systems (Fig. 2). It is cost-effective and straightforward to generate germ-free *Drosophila* [77] which, if required, can be maintained as a vigorous culture over multiple generations. Furthermore, germ-free *Drosophila* can be re-conventionalized with a standardized microbiota to study the role of specific microorganisms. *Drosophila* presents a repertoire of behaviors and many of these could be used to study core features of autism, like anxiety [79], social interactions [80], and light-pulse habituation [81] (Fig. 2). Autism-related genes and microbiome may have an impact on other body organs and may lead to comorbidities, such as GI issues [36], physical or craniofacial abnormalities [82], epilepsy [83], and other learning disabilities [84], which can also be studied in *Drosophila* using robust assays (Fig. 2).

Given that key components of conserved signaling pathways like Notch, Hedgehog, Wnt, and epidermal growth factor receptor (EGFR) were identified using *Drosophila* wings models [85–91], *Drosophila* wing provides an effective system to evaluate the role of autism and other neurodevelopment-related genes in identifying a signaling pathway that could be affected during development [92]. Interestingly, changes in GM by antibiotic treatment or enrichment of *Wolbachia* can induce morphological changes in the *Drosophila* wing [93, 94].

## The *Drosophila* gut shares a similar structure to the mammalian gut

The *Drosophila* gut is subdivided into the foregut, midgut, and hindgut, which broadly represent the esophagus, small intestine, and large intestine in mammals (Fig. 2). The *Drosophila* gut is composed of an epithelial layer surrounded by visceral muscles, nerves, and the trachea (Fig. 2). In mammals, intestinal epithelium is separated from the external environment by a mucus membrane. However, in *Drosophila*, intestinal epithelium is separated from the external environment by a peritrophic membrane, along with a thin mucus layer (Fig. 2D, E) [95].

The *Drosophila* intestinal epithelial layer is a complex tissue composed of various cell types and performs diverse functions, including digestion, hormone secretion and nutrient absorption. The intestinal epithelium is broadly made up of two types of differentiated cells: enterocytes (ECs) and enteroendocrine (EE) cells, as well as a small percentage of pluripotent intestinal stem cells (ISCs) and enteroblasts (EBs).

**Fig. 3:**
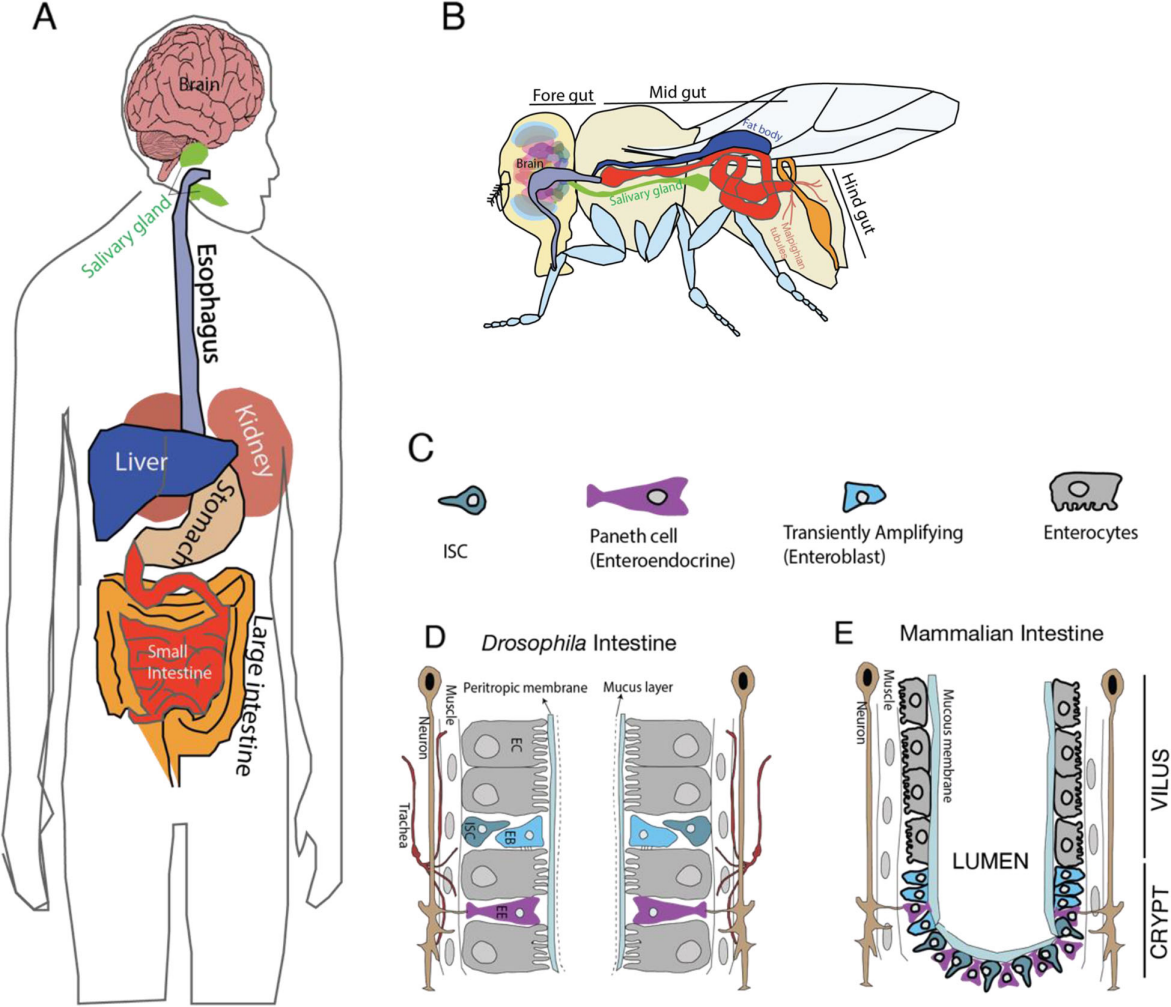
Illustration of *Drosophila* and human anatomical structures. **A**, **B** The gastrointestinal system in humans and flies is functionally analogous, shares a similar overall organizational structure, and performs both secretory and absorptive functions. **C** The intestinal epithelium of both humans and flies consists of differentiated epithelial cells, secretory enterocytes (ECs), and absorptive enteroendocrine (EE) cells. Colors are coded for similar structures. **D**, **E** *Drosophila* and mammalian intestines are composed of similar cell types with minor differences (adapted from https://droso4schools.wordpress.com/organs/ and [96])

The ECs secrete digestive enzymes and absorb nutrients, whereas the EE cells secrete hormones and regulate gut motility. Similar to mammals, the *Drosophila* intestine is a highly regenerative organ. The epithelial layer is renewed every 5–10 days through proliferation and differentiation of pluripotent ISCs to maintain homeostasis and regeneration. To maintain homeostasis, ISCs proliferate and give rise to a transient progenitor, the EB [equivalent to mammalian Transiently Amplifying (TA) cells]. In mammals, the gut epithelial layer comprises ECs and EEs (tuft, goblet, and paneth cells) confined to the villus, and ISCs and TAs are confined to crypts. Similar to the mammalian mucous layer, the *Drosophila* gut inner wall is further enveloped by a chitinous layer known as the peritrophic membrane, which serves as a protective barrier between the gut lumen and epithelial cells. The microbiota in the gut interacts with gut proteins, and changes in mucus membrane function may alter the microbial composition. The microbiota can interact with proteins in the peritrophic membrane and mucus membrane, in this way, can modify membrane’s properties to allow the passage of microbiota or derived metabolites through the epithelial wall (Fig. 3).

## ISC stem cell physiology is governed by conserved molecular mechanisms in *Drosophila* and mammals

**Fig. 4:**
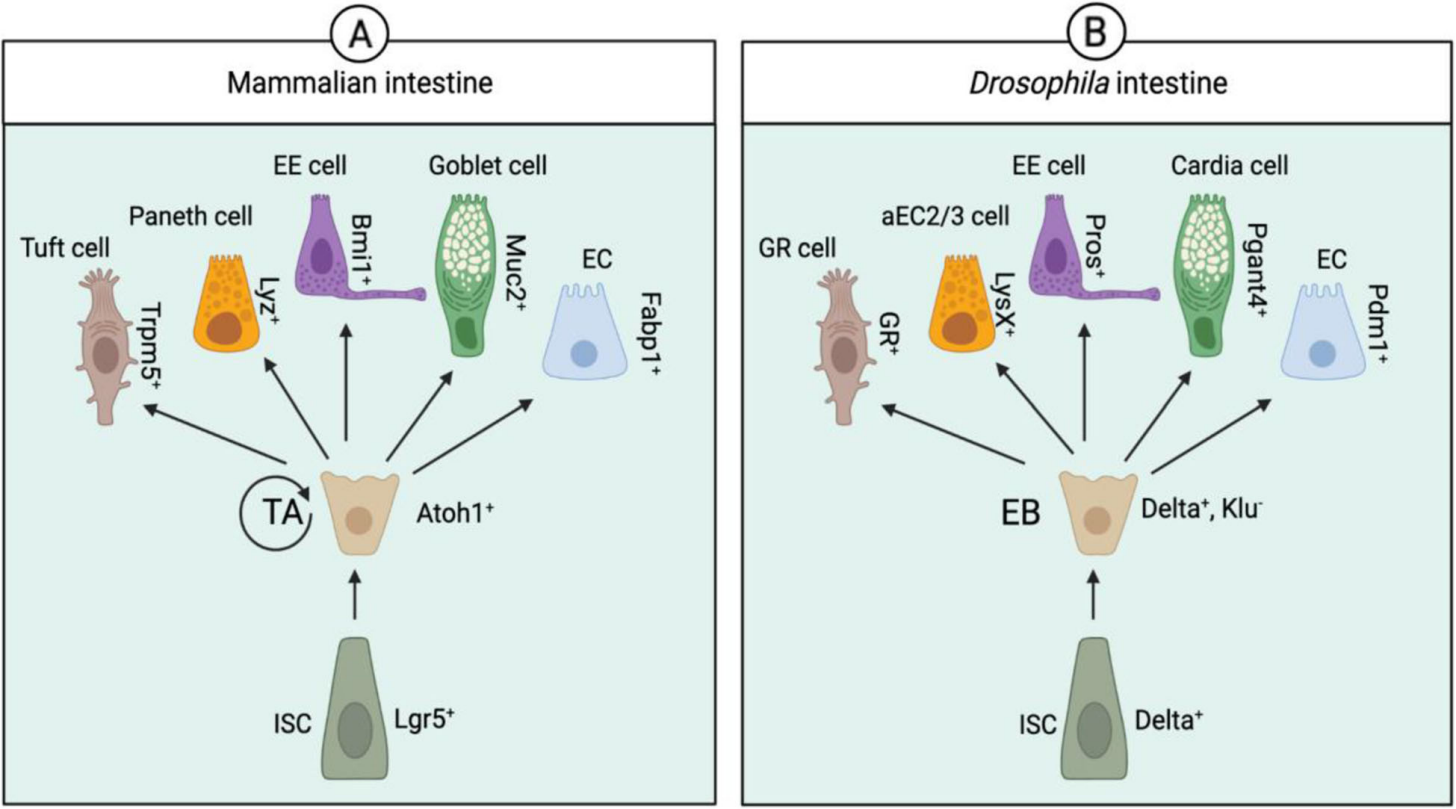
Schematic depicting cells of intestinal lineages in humans and *Drosophila*. The pathways of stem cell maintenance and differentiation are largely conserved between *Drosophila* and mammals with minor differences. Gut plasticity and homeostasis are maintained by regeneration of the gut epithelium. Intestinal stem cells (ISCs) divide asymmetrically to form transient amplifying (TA) cells in humans and enteroblasts (EB) in *Drosophila*, which differentiates to ECs and EEs. The ECs and EEs cells in the mammalian gut can further be divided into goblet cell (secrets mucous), paneth cell (secrets lysozymes), and tuft cell (expresses chemosensory receptors). In the *Drosophila* intestine, cardia cells produce mucus layer (analogous to mammalian goblet cell), aEC2/3 cells release lysozyme (analogous to mammalian paneth cell), and gustatory receptor-expressing cells in proventriculus (PV) are analogous to mammalian tuft cells. Intestinal cell types and their markers in mammalian and *Drosophila* intestines are shown (only one marker for each cell type has been shown). Trmp5 (Transient Receptor Potential Cation Channel Subfamily M Member 5), Lyz (Lysozyme), Enteroendocrine (EE), Bmi1 (B lymphoma Mo-MLV insertion region 1 homolog), Muc2 (Mucin 2), Enterocyte (EC), Fabp1 (Fatty Acid Binding Protein 1), TA (Transiently Amplifying), Atoh1 (Atonal BHLH Transcription Factor 1), ISC (Intestinal Stem Cell), Lgr5 (Leu-rich repeat-containing G protein-coupled receptor 5), GR (Gustatory Receptor), LysX (Lysozyme X), Pros (Prospero), Pgant4 (Polypeptide N-Acetylgalactosaminyltransferase 4), Pdm1 (POU domain protein 1), and Klu (Klumpfuss). Schematics are to show diversity in intestinal cell types and to highlight similarities between mammalian and *Drosophila* model systems; neither shape, size, nor color has depicted the real cells

Recent single-cell RNA sequencing (scRNA-Seq) analysis of mammalian and *Drosophila* intestines identified evolutionarily conserved gene signatures of gut cell types [97]. Using these signature genes and cluster analyses, several new cell types in the *Drosophila* intestine have been identified or proposed (Fig. 4). The molecular pathways largely governing the stem cell homeostasis in the gut are well conserved from *Drosophila* to humans. Molecular pathways regulating stem cell homeostasis in the gut are largely conserved throughout evolution, albeit with minor differences in ISC self-renewal and differentiation into TAs. In contrast, TA differentiation into ECs and EE cells follows a similar lineage system in mammals and *Drosophila* (Fig. 4) and is governed by similar molecular mechanisms with few differences. For example, Notch, Wnt, and BMP signaling determine the fate of proliferating cells in both *Drosophila* and mammals [98, 99]. Similarly, type II neuroblasts (NBs) in the *Drosophila* brain produce intermediate amplifying cells that closely resemble mammalian neural stem cells (NSCs) [100].

In mammals, paneth and TA cells are specifically found in specialized structures known as crypts. In the *Drosophila* intestine, which lacks crypts, proliferating stem cells are mainly confined to the midgut [101, 102]. *Drosophila* EEs are like mammalian EEs, tuft, and goblet cells [97]. Tuft cells are chemosensory, and *Drosophila* EEs (subpopulation of NPF, DH31 expressing cells) express gustatory chemosensory receptors [103]. *Drosophila* cardia cells (also known as secretory cells) are like mammalian goblet cells, consistent with their role in secreting mucins. The cardia cells in *Drosophila* synthesize and secrete the peritrophic membrane [103]. These cells are present in the proventriculus (PV) and express the enzyme O-glycosyltransferase (Pgant4). *Drosophila* enzyme Pgant4 is most like the mammalian ppGalNAc-T10, an abundant enzyme in the digestive system and regulates mucin secretion via mucin-type O-glycosylation [104]. The mammalian paneth cells are like *Drosophila* aEC2 and aEC3 (subsets of EC), consistent with their role in secreting lysozymes (which function in defense against bacteria) in both cell types [97] (Fig. 4).

Although *Drosophila* NBs and ISCs originate from distinct germ layers, pupal ISCs and Type I NBs share many similarities, e.g., both ISCs and type I NBs express delta (Dl) relative to their neighboring cells [105]. Moreover, several neural progenitor-specific transcription factors also regulate ISC maintenance and differentiation [106]. These studies suggest that similar molecular mechanisms might regulate ISC and NSC proliferation and differentiation. Therefore using *Drosophila* as a model to study gut or brain development, physiology and plasticity in relation to GUMBA is a good strategy.

## Many ASD-associated genes are highly conserved in *Drosophila* and are expressed in both the gut and the brain

*Drosophila* shares a high degree of genetic homology with mammals and exhibits conceptually analogous physiological and pathological mechanisms to higher organisms [66, 67]. *Drosophila* is often used to study the molecular mechanisms regulating gut homeostasis, plasticity, and behavior [107]. The *Drosophila* gut expresses many of the same proteins as the human gut [108]. We analyzed the expression enrichment score of high confidence category 1 ASD genes (https://gene.sfari.org/about-gene-scoring/). Each of these category 1 genes has been implicated in ASD and typically possesses at least three de novo likely gene-disrupting mutations [61].

Out of 207 high confidence autism-related genes, 203 (98%) were found to have an ortholog in fruit flies (Supplementary File). For 12 high confidence ASD genes (ALDH5A1, CACNA2D3, CSDE1, CSNK2A1, DYNC1H1, HDLBP, MED13, PSMD12, SMARCC2, SPAST, STXBP1, WDFY3), *Drosophila* had a perfect ortholog DIOPT score (orthology score was determined via flybase.org with 1 representing perfect orthology), while 141 genes had orthology score of ≥ 0.6 (confirmed by nine out of 15 databases), suggesting that ASD-related mechanisms involving these high confidence ortholog genes can be studied in *Drosophila* with high confidence. Fly transgenics expressing cDNA of human autism genes are also available from stock centers for 37 human ASD-related genes (Table 1). These transgenic lines can directly be used to prepare humanized *Drosophila* autism models or could be used in combination with corresponding fly gene mutants to rescue ASD-related phenotype and to test the functional effect of an ASD-linked genetic variant in those genes.

**Table 1:**
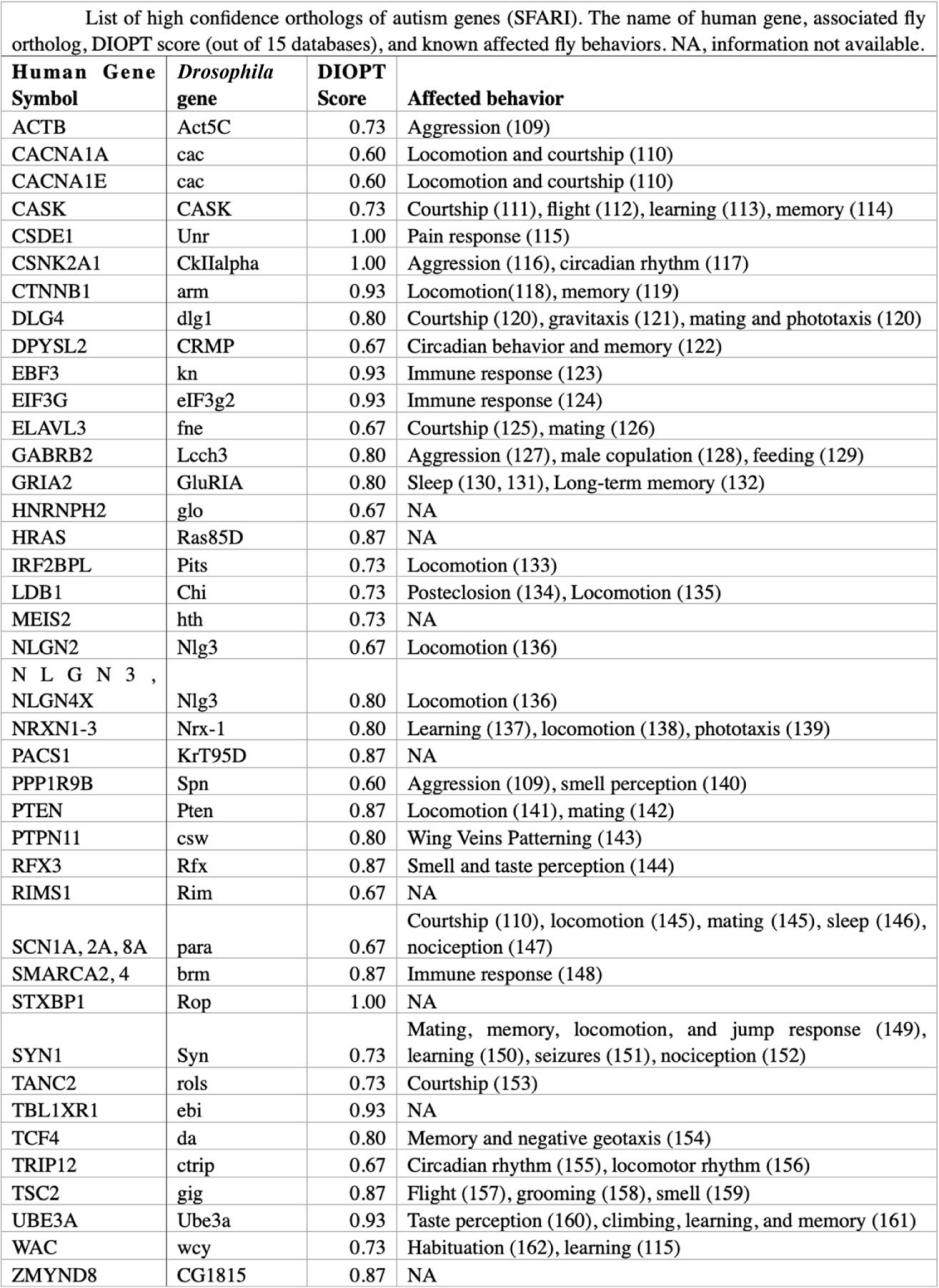
List of high confidence orthologs of autism genes (SFARI) [109–162]

Most of the identified genetic mutations (frameshift, nonsense, and splice-site) associated with ASD have mainly been related to the loss-of-function of the affected genes [163, 164]; missense mutations are more likely to have gain-of-function effects [165]. However, the impact of missense mutations mediated gain-of-function has recently been identified. For example, Pinggera et al. identified and validated a missense mutation V401L (valine to leucine at residue 401) as a gain-of-function mutation in one of the autism risk genes, CACNA1D [166], which may explain psychiatric and epileptic phenotypes observed in patients with this mutation. Similarly, gain-of-function missense mutation A636T (alanine to threonine amino acid replacement at residue 636 ) in Glutamate Ionotropic Receptor AMPA Type Subunit 1 (*GRIA1*) leads to a constitutively active channel [167] and may be linked to some ASD features like intellectual disability (ID), delayed language development and delayed motor development [168]. Similarly, in a *Drosophila* model of Angelman syndrome (an autism-related disorder), both overexpression and loss of fly UBE3A activity resulted in similar behavioral defects [161] as presumably both genetic knockdown and overexpression resulted in accumulation or too little UBE3A substrate, respectively, which have similar consequences and behavioral effects. Collectively, these studies suggest that apart from the loss-of-function studies, more studies are required to gain insight on specific missense mutations causing gain-of-function phenotypes.

The *Drosophila* model provides a robust system to evaluate both loss- and gain-of-function roles of these human ASD risk genes listed in Table 1. The wild-type human cDNAs could be expressed in either intestine or brain using specific Gal4 drivers. Their either sole impact or rescue effect, combined with fly ortholog mutant, on the microbiome, gut physiology, and plasticity and fly behaviors, can be studied. For many of these genes, Gal4 insertion lines are also available from stock centers. These Gal4 lines are an important resource to delineate neuronal identity or functional neuronal circuits involved in regulating behaviors related to ASD.

**Fig. 5:**
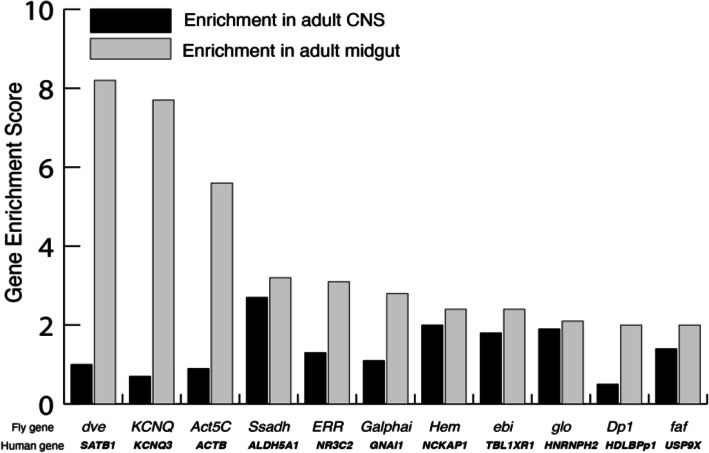
Top ASD-related genes highly enriched in *Drosophila* gut. Among genes with moderate orthology score > 0.6, 11 genes have higher expression in the gut than in the brain and enrichment of at least 200% compared to the whole body. Enrichment scores were obtained from http://flyatlas.gla.ac.uk/FlyAtlas2

Out of the 141 genes with a moderate orthology score of at least 0.6, 116 (82%) genes were enriched in the adult brain. Enrichment scores were obtained using Flyatlas2.0, http://flyatlas.gla.ac.uk/FlyAtlas2. We considered a gene to be enriched in a particular tissue if its expression was at least 50% higher in the tissue than whole-body expression (Supplementary Table 1). Among these 141 genes, 94 genes were enriched in the adult *Drosophila* brain and gut, 22 were enriched only in the brain, and 78 were more enriched in the gut than in the brain. Among these 78 gut-brain-expressing genes, 11 genes were enriched in the gut by 200% or more (Fig. 5). We propose that these 11 genes are good candidates to study GUMBA in relation to ASD in *Drosophila*. Among these genes, we did not find any gene that exclusively expresses in the gut.

## Limitations of *Drosophila* GUMBA system

Although *Drosophila* genetic tool kits provide access to the advanced reagents required to dissect the role of most human orthologs, not all ASD genes can be modeled in *Drosophila* since not all ASD genes have an ortholog in *Drosophila*. Additionally, GM in *Drosophila* is very simple which limits the studies on complex host-microbiota interaction. Although *Drosophila* can be humanized with human microbiota, *Drosophila* gut is not suitable for the growth of anaerobic microbiota, which are dominating microorganisms in the human gut. The relevance of *Drosophila* GM studies to humans is limited to the standard microbiome like *Lactobacillus* sp., which are common between humans and flies*.* Furthermore, *Drosophila* lacks adaptive immunity and has a very simple innate immunity, making it challenging to recapitulate complex changes in the immune system that might take place following GM-mediated changes in ASD pathophysiology.

## Conclusion and future directions

Through animal model-based studies, primarily rodent-based research, our understanding of the effects of the microbiome, its interaction between gut and brain, and its effects on animal physiology and disease pathology has significantly been improved. Given the genetic and neurobehavioral advantages of *Drosophila*, models of ASD can be generated by studying ASD relevant phenotypes in mutant or in flies with genetically knockdown orthologs of ASD-related genes and their association with the GM, genes showing effects can further be validated by expressing cDNA of human ASD genes. *Drosophila* lines that are germ-free wild type, overexpressing ASD-related genes, or deficient of ASD-related genes can be exposed to GM from human ASD patients to determine whether the GM regulates ASD-associated behaviors. Apart from core ASD features, *Drosophila* can also be readily used to study ASD comorbidities or non-neuronal development defects like craniofacial features. Furthermore, highly advanced techniques like optogenetics, live imaging, and neural circuit mapping can be used to gain mechanistic insight and a better understanding of ASD-associated neural circuits and GUMBA components. These approaches will facilitate the identification of better therapeutics for ASD.

## Supplementary Information

**Additional file 1: Supplementary Table 1.** List of 141 orthologs related to ASD high confidence list with their DIOPT scores.

## Data Availability

The datasets analyzed during the current study are included in this review.
