# Supplementary material for: The gut-microbiota-brain axis in autism: what Drosophila models can offer?
Source: J Neurodev Disord. 2021 Sep 15;13:37. doi: 10.1186/s11689-021-09378-x (PMC8442445; doi:10.1186/s11689-021-09378-x)
Supplement: Supplementary file 1 — Additional file 1: Supplementary Table 1. List of 141 orthologs related to ASD high confidence list with their DIOPT scores. [file 11689_2021_9378_MOESM1_ESM.docx]

**Supplementary Table 1. List of 141 orthologs related to ASD high confidence list with their DIOPT scores.**

| **GENE SYMBOL** | ***Drosophila* ortholog** | **DIOPT Score** |
| --- | --- | --- |
| **ACTB** | **Act5C** | **0.73** |
| **ADSL** | **AdSL** | **0.93** |
| **AFF2** | **lilli** | **0.60** |
| **ALDH5A1** | **Ssadh** | **1.00** |
| **AP2S1** | **AP-2sigma** | **0.93** |
| **ARID1B** | **osa** | **0.80** |
| **ASH1L** | **ash1** | **0.67** |
| **ATRX** | **XNP** | **0.67** |
| **BAZ2B** | **tou** | **0.67** |
| **BCL11A** | **CG9650** | **0.73** |
| **BRAF** | **Raf** | **0.93** |
| **BRSK2** | **sff** | **0.80** |
| **CACNA1A** | **cac** | **0.60** |
| **CACNA1C** | **Ca-alpha1D** | **0.80** |
| **CACNA1E** | **cac** | **0.60** |
| **CACNA2D3** | **stj** | **1.00** |
| **CAPRIN1** | **Capr** | **0.60** |
| **CASK** | **CASK** | **0.73** |
| **CELF4** | **bru3** | **0.67** |
| **CHD2** | **Chd1** | **0.87** |
| **CHD3** | **Mi-2** | **0.80** |
| **CHD7** | **kis** | **0.67** |
| **CHD8** | **kis** | **0.67** |
| **CIC** | **cic** | **0.80** |
| **CNOT3** | **Not3** | **0.67** |
| **CORO1A** | **coro** | **0.87** |
| **CREBBP** | **nej** | **0.80** |
| **CSDE1** | **Unr** | **1.00** |
| **CSNK2A1** | **CkIIalpha** | **1.00** |
| **CTCF** | **CTCF** | **0.60** |
| **CTNNB1** | **arm** | **0.93** |
| **CUL3** | **Cul3** | **0.87** |
| **DDX3X** | **bel** | **0.87** |
| **DEAF1** | **Deaf1** | **0.80** |
| **DIP2A** | **DIP2** | **0.87** |
| **DLG4** | **dlg1** | **0.80** |
| **DPYSL2** | **CRMP** | **0.67** |
| **DSCAM** | **Dscam4** | **0.87** |
| **DYNC1H1** | **Dhc64C** | **1.00** |
| **DYRK1A** | **mnb** | **0.67** |
| **EBF3** | **kn** | **0.93** |
| **EHMT1** | **G9a** | **0.73** |
| **EIF3G** | **eIF3g2** | **0.93** |
| **ELAVL3** | **fne** | **0.67** |
| **EP300** | **nej** | **0.80** |
| **FMR1** | **Fmr1** | **0.73** |
| **FOXG1** | **slp2** | **0.60** |
| **GABRB2** | **Lcch3** | **0.80** |
| **GABRB3** | **Lcch3** | **0.87** |
| **GIGYF1** | **Gyf** | **0.60** |
| **GIGYF2** | **Gyf** | **0.67** |
| **GNAI1** | **Galphai** | **0.80** |
| **GRIA2** | **GluRIA** | **0.80** |
| **GRIN2B** | **Nmdar2** | **0.73** |
| **HDLBP** | **Dp1** | **1.00** |
| **HNRNPH2** | **glo** | **0.67** |
| **HNRNPU** | **CG30122** | **0.67** |
| **HRAS** | **Ras85D** | **0.87** |
| **IQSEC2** | **siz** | **0.80** |
| **IRF2BPL** | **Pits** | **0.73** |
| **KANSL1** | **nsl1** | **0.60** |
| **KATNAL2** | **CG10793** | **0.67** |
| **KDM3B** | **Kdm3** | **0.67** |
| **KDM5B** | **Kdm5** | **0.87** |
| **KDM5C** | **Kdm5** | **0.87** |
| **KDM6B** | **Utx** | **0.60** |
| **KMT2C** | **trr** | **0.67** |
| **LDB1** | **Chi** | **0.73** |
| **LZTR1** | **Lztr1** | **0.87** |
| **MBOAT7** | **frj** | **0.93** |
| **MED13** | **skd** | **1.00** |
| **MED13L** | **skd** | **0.80** |
| **MEF2C** | **Mef2** | **0.73** |
| **MEIS2** | **hth** | **0.73** |
| **MKX** | **CG11617** | **0.73** |
| **NAA15** | **Naa15-16** | **0.87** |
| **NBEA** | **rg** | **0.80** |
| **NCKAP1** | **Hem** | **0.93** |
| **NF1** | **Nf1** | **0.87** |
| **NIPBL** | **Nipped-B** | **0.80** |
| **NLGN2** | **Nlg3** | **0.67** |
| **NLGN3** | **Nlg3** | **0.80** |
| **NLGN4X** | **Nlg3** | **0.73** |
| **NR4A2** | **Hr38** | **0.87** |
| **NRXN1** | **Nrx-1** | **0.80** |
| **NRXN2** | **Nrx-1** | **0.80** |
| **NRXN3** | **Nrx-1** | **0.73** |
| **NSD1** | **NSD** | **0.67** |
| **NUP155** | **Nup154** | **0.93** |
| **PACS1** | **KrT95D** | **0.87** |
| **PAH** | **Hn** | **0.93** |
| **PHF12** | **CG3815** | **0.93** |
| **PHIP** | **BRWD3** | **0.73** |
| **PPP1R9B** | **Spn** | **0.60** |
| **PPP2R5D** | **wrd** | **0.87** |
| **PPP5C** | **PpD3** | **0.93** |
| **PSMD12** | **Rpn5** | **1.00** |
| **PTEN** | **Pten** | **0.87** |
| **PTK7** | **otk** | **0.93** |
| **PTPN11** | **csw** | **0.80** |
| **RERE** | **Gug** | **0.67** |
| **RFX3** | **Rfx** | **0.87** |
| **RIMS1** | **Rim** | **0.67** |
| **RORB** | **Hr3** | **0.73** |
| **SCN1A** | **para** | **0.67** |
| **SCN2A** | **para** | **0.73** |
| **SCN8A** | **para** | **0.80** |
| **SETD1A** | **Set1** | **0.73** |
| **SETD2** | **Set2** | **0.73** |
| **SHANK2** | **Prosap** | **0.67** |
| **SIN3A** | **Sin3A** | **0.87** |
| **SLC6A1** | **Gat** | **0.93** |
| **SLC9A6** | **Nhe3** | **0.87** |
| **SMARCA2** | **brm** | **0.87** |
| **SMARCA4** | **brm** | **0.80** |
| **SMARCC2** | **mor** | **1.00** |
| **SOX5** | **Sox102F** | **0.73** |
| **SPAST** | **spas** | **1.00** |
| **SRPRA** | **SrpRalpha** | **0.93** |
| **STXBP1** | **Rop** | **1.00** |
| **SYN1** | **Syn** | **0.73** |
| **SYNGAP1** | **raskol** | **0.60** |
| **TANC2** | **rols** | **0.73** |
| **TAOK1** | **Tao** | **0.80** |
| **TBCK** | **CG4041** | **0.93** |
| **TBL1XR1** | **ebi** | **0.93** |
| **TCF4** | **da** | **0.80** |
| **TLK2** | **Tlk** | **0.73** |
| **TM9SF4** | **TM9SF4** | **0.93** |
| **TRIO** | **trio** | **0.80** |
| **TRIP12** | **ctrip** | **0.67** |
| **TSC1** | **Tsc1** | **0.87** |
| **TSC2** | **gig** | **0.87** |
| **UBE3A** | **Ube3a** | **0.93** |
| **UBR1** | **Ubr1** | **0.80** |
| **UPF3B** | **Upf3** | **0.73** |
| **USP9X** | **faf** | **0.93** |
| **VPS13B** | **Vps13B** | **0.73** |
| **WAC** | **wcy** | **0.73** |
| **WDFY3** | **bchs** | **1.00** |
| **ZMYND8** | **CG1815** | **0.87** |
